# Trajectories of alcohol screening and brief intervention (ASBI) performance and their associations with long-term performance and alcohol use outcomes: an observational study in a large US integrated healthcare delivery system

**DOI:** 10.1186/s43058-025-00842-6

**Published:** 2025-12-26

**Authors:** Felicia W. Chi, Yun Lu, Vanessa A. Palzes, Thekla B. Ross, Constance Weisner, Joseph Elson, Verena E. Metz, Stacy A. Sterling

**Affiliations:** 1 Division of Research, Kaiser Permanente Northern California, 4480 Hacienda Drive, Pleasanton, CA 94588 USA; 2 The Permanente Medical Group, 1600 Owens Street, San Francisco, CA 94158 USA; 3 Department of Psychiatry and Behavioral Sciences, University of California, San Francisco, 675 18th St., San Francisco, CA 94143 USA; 4 Department of Health Systems Science, Kaiser Permanente Bernard J. Tyson School of Medicine, 98 S Los Robles Ave, Pasadena, CA 91101 USA

**Keywords:** Alcohol screening and brief intervention, Implementation performance trajectories, Long-term sustainment, Population-level drinking outcomes

## Abstract

**Background:**

Unhealthy alcohol use is a public health problem with significant health, social and economic impacts. Alcohol screening and brief intervention (ASBI) in adult primary care is an evidence-based approach enabling early identification and intervention of unhealthy alcohol use. However, large-scale implementation and sustainment of ASBI in routine clinical practice remains a challenge, and little is known about its population-level impact. Using electronic health record (EHR) data in a large integrated healthcare system in Northern California that implemented systematic ASBI in adult primary care in mid-2013, this observational study examined: 1) trajectories of ASBI performance over 5 years post systematic implementation, and 2) their associations with both later ASBI performance and alcohol use outcomes.

**Methods:**

Using the health plan’s EHR data, we calculated annual screening rates of adults with a primary care visit, and brief intervention (BI) rates among those with a positive screen (i.e., reporting alcohol consumption exceeding the age and sex specific daily and weekly low-risk National Institute on Alcohol Abuse and Alcoholism guidelines), for 57 medical facilities from years 2014 to 2021. We conducted latent class growth analysis using annual screening and BI rates to characterize trajectories of ASBI performance from years 2014–2018. Multivariable mixed-effects models were fit to examine the associations of ASBI performance trajectories with later ASBI performance and facility-level alcohol use outcomes.

**Results:**

Three distinct screening performance trajectory groups (low-, middle- and high-performance) and four distinct BI performance trajectory groups (low-, improving-, middle- and high-performance) were identified. Facilities in the low-BI-performance group had panels of patients living in more deprived neighborhoods compared to the other 3 BI performance groups. After accounting for repeated measures and adjusting for time and patient panel characteristics, we found that screening and BI performance trajectories during 2014–2018 were significantly associated with screening and BI rates 2019–2021, respectively. We also observed a steeper decline in percentages reporting “exceeding daily drinking limits” and “having 5 + binge drinking days” over time among patients of facilities in the improving- and high-BI-performance groups.

**Conclusions:**

Early success in ASBI performance is associated with long-term sustainability and may be associated with long-term population-level drinking outcomes.

**Supplementary Information:**

The online version contains supplementary material available at 10.1186/s43058-025-00842-6.

Contributions to the literature

- Sustained and systematic implementation of alcohol screening and brief intervention (ASBI) in primary care has proven challenging even after decades of effectiveness evidence and expert recommendation.
- Ours is among the first large-scale population-based studies of ASBI performance trajectories over 5 years post-implementation in adult primary care in a large healthcare system, and the associations with long-term sustainment and alcohol use outcomes at population level over time.
- Findings underscore the importance of early ASBI implementation efforts to long-term sustainment, identify patient panel characteristics associated with performance of ASBI implementation and sustainment; and inform the need for future ASBI implementation research.

## Background

Unhealthy alcohol use, encompassing a range of alcohol use from risky drinking exceeding recommended daily and weekly drinking limits to alcohol use disorder, is a serious public health concern which contributes substantially to mortality and morbidity. The World Health Organization reports that unhealthy alcohol use constitutes over 5% of the global burden of disease and leads to 3 million deaths annually [1]. While estimates vary, a third of U.S. adults may exceed low-risk alcohol consumption guidelines [2]. It is also a common problem experienced by adult primary care patients but one that is, unfortunately, under-identified and under-treated [3, 4].

Alcohol screening and brief intervention (ASBI) is an effective approach to address unhealthy alcohol use across medical settings, including primary care [5–9]. This approach to identification and early intervention for risky alcohol use is non-confrontational, involving systematic screening using evidence-based instruments, a brief intervention (BI) including a systematic protocol for referral to specialty substance use treatment for those who need it. While BI may vary in how it is delivered, it is typically informed by Motivational Interviewing, a therapeutic approach used to address a wide variety of behavioral health problems, but most often alcohol use [10]. BIs are intended to increase patients’ intrinsic motivation to change risky behavior through consideration of the pros and cons of such behaviors. ASBI is endorsed by the National Institutes of Health, the Department of Health and Human Services, the U.S. Preventive Services Task Force, and the World Health Organization [11–14]. However, after decades of policy recommendations and efforts to implement it in routine clinical practice, as is the case for many preventive services, widespread implementation of ASBI in primary care is still rare, except in a few large, integrated healthcare systems such as the Veterans Health Administration [15] and Kaiser Permanente [16] in the U.S. and in Scotland [17]. Further, sustainment remains a challenge, due to various factors at patient- (e.g., stigma, language barrier), provider- (e.g., time constraints, lack of training), program- (e.g., lack of continuous technical support or performance monitoring) and system-levels (e.g., limited financial resources, lack of leadership support) [18, 19]. In addition, little is known about the population-level impact of ASBI implementation and sustainment in real-world settings.

Using electronic health record (EHR) data from Kaiser Permanente Northern California (KPNC), a large United States’ integrated healthcare system that implemented systematic ASBI in adult primary care in June 2013, this observational study examined: 1) trajectories of ASBI performance over 5 years post systematic implementation, and 2) their associations with long-term ASBI performance in years 6–8. We also conducted exploratory analyses to examine associations of ASBI performance over 5 years post-implementation with alcohol use outcomes over time. With KPNC’s sustained ASBI program and robust, longitudinal EHR data, this study leverages a unique opportunity to retrospectively evaluate ASBI performance trajectories across different phases over 8 years and explore the population-level impact of ASBI implementation and sustainment. ASBI performance denotes the rates at which adult primary care clinics carry out screening and brief intervention protocols.

## Methods

### Study design

This is a longitudinal, observational study evaluating ASBI performance across two phases: implementation and short-term sustainment (first 5 years post-implementation), and long-term sustainment (after first 5 years post-implementation), for KPNC’s medical facilities between years 2014 and 2021. Analysis was conducted at the medical facility level using annual ASBI rates during the study period.

### Study site

Kaiser Permanente Northern California (KPNC) is a large, non-profit, integrated healthcare delivery system serving over 4.5 million members. Its membership is socio-economically diverse and highly representative of the statewide [20] and U.S. populations with access to care [21]. It provides care via employer-based plans, Medicare, Medicaid and insurance exchanges, and psychiatry and addiction treatment as a covered benefit. The region has 21 hospitals, 207 medical offices, and ~ 2,800 physicians and providers in Adult Medicine and Family Medicine; all of these primary care departments were included in this study.

In June 2013, KPNC implemented systematic ASBI into its adult (18 +) primary care workflow. Using National Institute on Alcohol Abuse and Alcoholism (NIAAA) evidence-based screening instruments embedded in the EHR, medical assistants ask a single-item question about heavy drinking (‘How many times in the past 3 months have you had five or more drinks in a day’ (for men aged 18–65 years), or ‘four or more drinks’ for men aged ≥ 66 years and women of all ages), followed by two questions on typical drinking days per week and typical number of drinks per drinking day [22]. Medical assistants ask these questions as they collect other vital sign information, and record patient answers in the EHR. A positive screening is defined as exceeding either the daily or weekly drinking limits per NIAAA recommendations (> 7 drinks/week for women of all ages and men aged 66 and older, or > 14 drinks/week for men aged 18–65). Brief intervention or referral to treatment is provided by physicians, as needed. See online supplemental document and Sterling et al. [23] for detailed descriptions of the protocol for the System-wide ASBI at KPNC Adult Primary Care. This study followed the REporting of studies Conducted using Observational Routinely-collected Data (RECORD) reporting guidelines [24] and was approved by the KPNC Institutional Review Board (IRB) with a waiver of informed consent.

### Measures

#### Annual screening and BI rates

Using KPNC EHR data, we calculated annual screening rates of adults with any primary care visit, and BI rates among those with a positive screen (i.e., exceeding the NIAAA age and sex specific daily and weekly low-risk guidelines), for each medical facility from 2014 to 2021.

The 8-year study period was divided into 2 phases: *implementation and short-term sustainment* (2014–2018, first 5 years post-implementation) and *long-term sustainment* (2019–2021, after first 5 years post-implementation). We characterized trajectories of ASBI performance during implementation and short-term sustainment phase and examined their associations with ASBI performance during long-term sustainment phase.

#### Facility-level alcohol use outcomes

Four facility-level alcohol use outcomes were generated for each of the years during 2014–2021 using KPNC EHR data:

1. Proportion exceeding the age and sex specific daily drinking limits (heavy episodic drinking” or “HED”)
2. Proportion having 5 + HED, an indicator of heightened risk of future severe alcohol use disorder [25]
3. Proportion exceeding the age and sex specific weekly drinking limits
4. Proportion exceeding both the daily and weekly limits

among adult members aged ≥ 18 years old who had a primary care visit to that facility and were screened for alcohol use in that year.

#### Facility-level patient panel characteristics

For each facility, we identified the patient panel for each of the study year then pulled patient panel characteristics including age, sex, race/ethnicity, neighborhood deprivation index (NDI) [26], insurance coverage (Medicaid or non-Medicaid), and clinical risk score (often used to measure clinical severity and predict clinical burden and clinic costs) during the current year based on age, sex, and diagnostic data from the past 12 months [27]. We then generated the annual summative measures of panel size; proportions of women, White, age ≥ 65, Medicaid insured; and average NDI and risk score among patient panel for each medical facility from 2014 to 2021. A binary indicator for each patient panel characteristic at each year was also created to indicate above/below median across facilities.

### Statistical analysis

All analyses were conducted using SAS 9.4. Two-sided *p*-value < 0.05 was considered statistically significant.

To characterize ASBI outcomes during implementation and short-term sustainment phases, we conducted latent class growth analysis (LCGA) using annual screening and brief intervention rates from years 2014–2018, i.e., a 5-year period post the systematic ASBI implementation, using data for the 57 facilities with data of ASBI rates for at least 2 years during 2014–2018. LCGA is a data-driven approach increasingly applied in medicine and epidemiology to identify subgroups with distinct trajectories of the outcomes of interest, enabling examination of ASBI performance based on common rates observed in the real-world settings over time, rather than based on rigid cut-offs that may not represent performance well. We used a SAS procedure based on mixture modeling for estimating developmental trajectories (PROC TRAJ) [28], thus allowing identification of distinct screening and BI rate trajectory classes during the time window examined. Facilities with missing data on ASBI rates can be included, because PROC TRAJ makes use of all available data to estimate trajectories to describe the sample, making case-wise deletion unnecessary. The model was estimated under the assumption of a varying (rather than fixed) number of latent classes. Choice for the optimal number of classes was guided by the Bayesian Information Criterion, the recommended and currently most-widely used criteria for model fit [29], and theoretical interpretability of the classes. All 57 facilities were then classified into their most probable screening and BI performance trajectory groups based on the posterior probabilities.

To examine how ASBI trajectories during implementation and short-term sustainment phase were associated with ASBI performance during long-term sustainment phase, we fit two multivariable mixed models to examine associations of screening and BI trajectory groups during 2014–2018 with annual screening and BI rates during 2019–2021; each model accounted for repeated measures (i.e., one facility can have up to 3 data points) while adjusting for time and patient panel characteristics.

We also conducted exploratory analyses on the associations of ASBI trajectories during implementation and short-term sustainment phase with alcohol use outcomes during 2014–2021. For each of the four alcohol use outcomes, we estimated the adjusted average prevalence rates across years by fitting two separate multivariable mixed models; each includes the main effect of screening (or BI) trajectory groups, time, and the interaction of the two, while adjusting for the patient panel characteristics.

## Results

For years 2014–2021, average screening rate at KPNC’s facilities was 91% in 2014, remained high up to 2019, decreased to 76% in 2020, then increased to 81% in 2021. Average BI rates among those with a positive screen was 40% in 2014, increased to 57% in 2017 and remained above 50% in 2018 and 2019, then decreased to 38% in 2020 and 2021. Yearly summative measures of average panel size; average % of women, White, age ≥ 65, Medicaid insured; and average NDI and risk score among patient panel across medical facilities were stable during the period. Please refer to our prior paper (Sterling et al. [23]) for details.

### Screening performance trajectories during implementation/short-term sustainment phase and associated patient panel characteristics

**Fig. 1:**
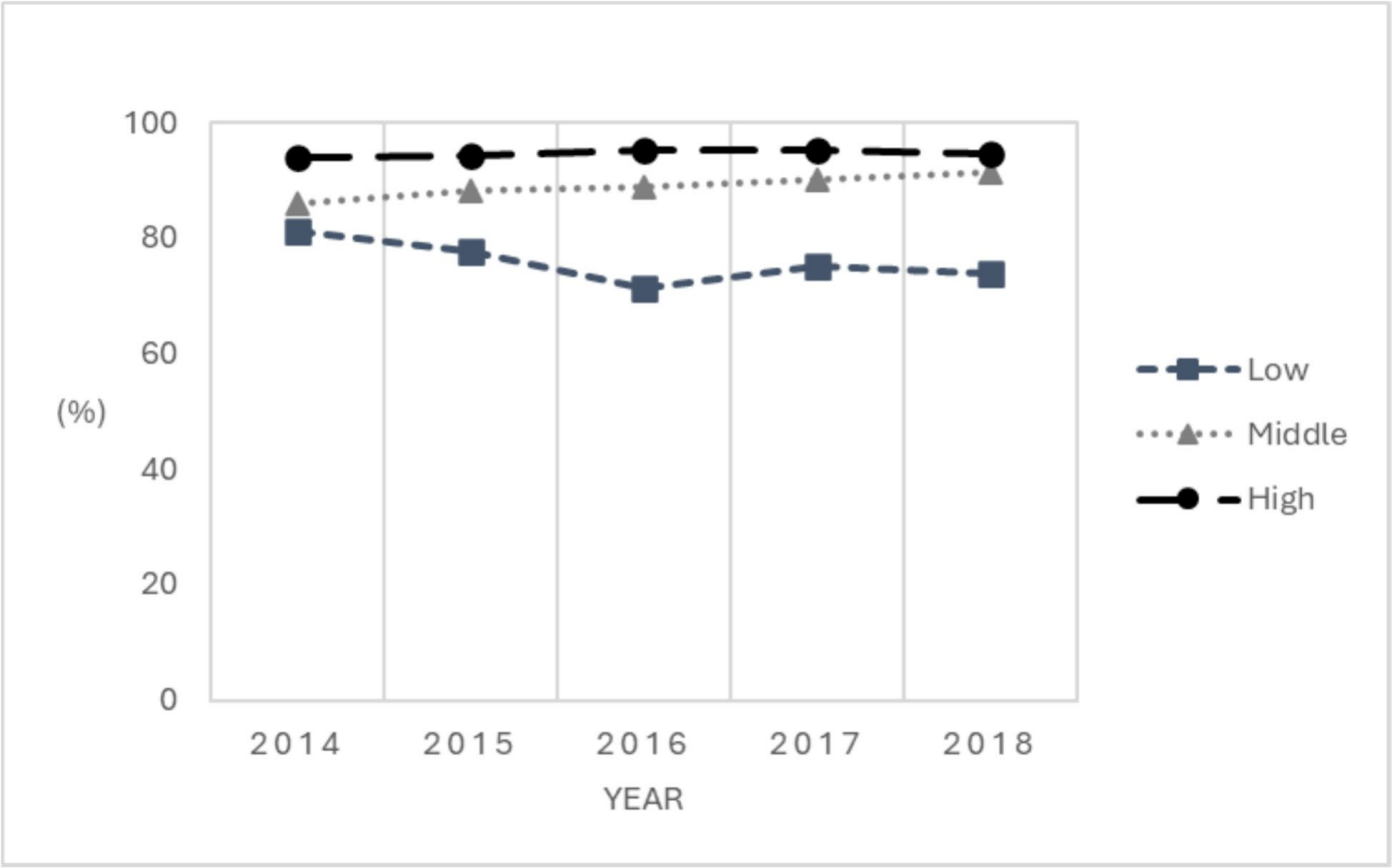
Screening Performance Groups, 2014–2018. Note. Following the LCGA, the annual screening rates 2014–2018 are represented by dots, allowing the visualization of their changing trends for the final 3 classes identified. LCGA = latent class growth analysis

LCGA was performed using data for the 57 facilities with at least 2 years during 2014–2018. After carefully comparing model fit indices and taking theoretical justification into account for each class solution, a three-class solution was determined as the optimal model for screening performance trajectories, as the three-class model had the smallest negative number (sTable 1 for summary of statistical fit across models). We classified all 57 facilities into the most probable screening groups for which they have the highest posterior probability of membership and examined the average posterior probabilities of group membership. In the probability matrix, diagonal values were closer to 1 and off-diagonal values closer to 0, suggesting a high degree of certainty (sTable 2). Figure 1 presents the three distinct screening performance trajectory groups, named as low, middle and high screening performance groups, respectively, for descriptive purpose.

Out of the 57 facilities, 36 (63.2%), 15 (26.3%) and 6 (10.5%) were classified into the high, middle and low screening performance groups, respectively. Screening rates during 2014–2018 were consistent for the high and middle groups, with rates ranging 94–95% and 86–91%, respectively. For the low screening performance group, the rate decreased from 81% in 2014 to 74% in 2018.

When examining associations between patient panel characteristics and screening performance during implementation and short-term sustainment phases (2014–2018), we found no significant differences in any of the patient panel characteristics examined across the screening performance groups (not shown).

### BI performance trajectories during implementation/short-term sustainment phase and associated patient panel characteristics

**Fig. 2:**
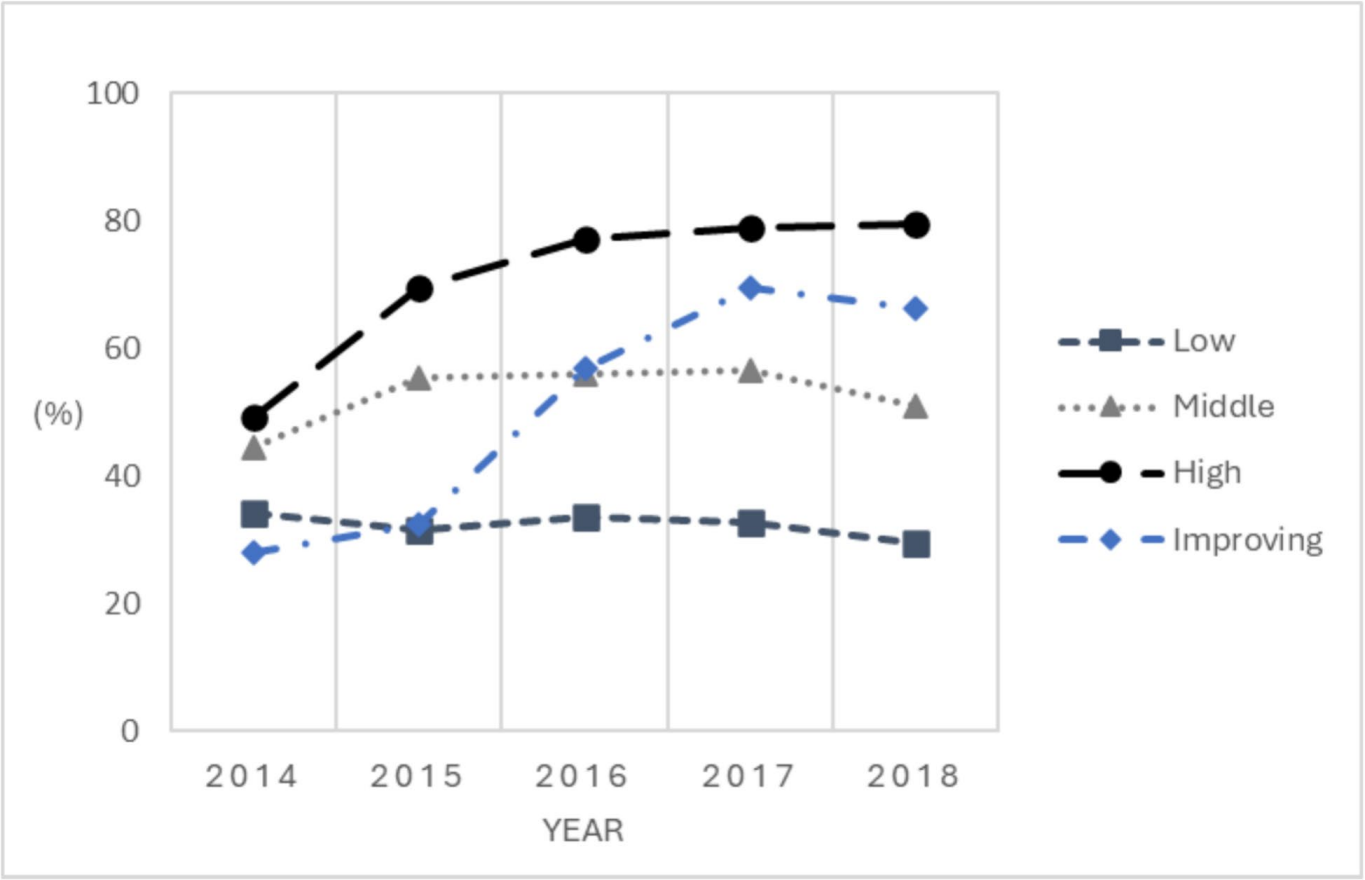
BI Performance Groups, 2014–2018. Note. Following the LCGA, the annual BI rates 2014–2018 are represented by dots, allowing the visualization of their changing trends for the final 4 classes identified. BI = brief intervention. LCGA = latent class growth analysis

Model fit indices suggested a three-class solution as best fit model solution for BI performance during 2014–2018, with the three-class model having the smallest negative number (sTable 3). However, the 4-group BI performance model suggested an additional “improving group,” the performance of which at beginning of the period close to the low performing group, and at end of the period close to the high performing group; all the facilities classified in this group had 5 data points 2014–2018, indicating that the trajectory observed was not driven by missing data. Further, this sub-group may provide valuable information with regard to important factors associated with improving BI performance. Thus, we kept the 4-group classification in the subsequent analyses. We examined the mean posterior probability of group membership and found strong separations among groups (sTable 4). Figure 2 presents the four distinct BI performance trajectory groups, named as low, improving, middle and high BI performance groups, respectively.

Out of the 57 facilities, 17 (29.8%), 13 (22.8%) 8 (14.0%) and 19 (33.3%) were classified into the high, middle, improving and low BI performance groups, respectively. For the high BI performance group, the BI rates increased from 49% in 2014 to 80% in 2018. For the improving group, the rates were low during the implementation phase (28% in 2014 and 33% in 2015), then increased from 57% in 2016 to 66% in 2018. For the middle and low BI performance groups, BI rates were relatively more consistent during 2014–2018, with rates ranging 46–57% for the middle BI group and 30–34% for the low BI group.

When examining associations between patient panel characteristics and BI performance during 2014–2018, we found significant differences in average NDI across BI groups during 2014–2018 (*p* = 0.002). As shown in Table 1, facilities in the Low BI performance group had panels of patients living in more deprived neighborhood contexts compared to the other 3 BI performance groups.

**Table 1:** Patient panel characteristics by BI performance groups during 2014–2018

	Low	Improving	Middle	High
	Mean (95% CI)	Mean (95% CI)	Mean (95% CI)	Mean (95% CI)
Panel Characteristics				
Proportion of age ≥ 65	17.2 (14.6, 19.9)	18.4 (14.3, 22.5)	15.4 (12.2, 18.7)	15.3 (12.5, 18.1)
Proportion of women	51.3 (50.2, 52.4)	52.3 (50.5, 54.0)	51.7 (50.3, 53.0)	52.2 (51.0, 53.4)
Proportion of White	41.7 (34.4, 49.1)	53.6 (42.3, 64.9)	38.9 (30.1, 47.8)	51.1 (43.4, 58.9)^d,f^
Proportion of Medicaid-insured	3.3 (2.2, 4.4)	3.4 (1.7, 5.2)	3.3 (2.0, 4.7)	3.1 (1.9, 4.3)
Average NDI	0.07 (−0.11, 0.25)	−0.40 (−0.68, −0.12)	−0.26 (−0.48, −0.04)	−0.41 (−0.61, −0.22)^a,b,c^
Average risk score	3.48 (3.22, 3.75)	3.35 (2.94, 3.76)	3.10 (2.78, 3.42)	3.03 (2.75, 3.31)^c^
Average panel size	2213 (1954, 2472)	2082 (1685, 2479)	2098 (1786, 2411)	2272 (1998, 2546)

For each panel characteristic, means and 95% confidence intervals by BI performance group were estimated from mixed-effect models accounting for non-independence of repeated measures over time within facilities

*BI* brief intervention, *CI* confidence intervals, *NDI* neighborhood deprivation index

^a,b,c,d,e,f^: significant *p* values for comparisons of low vs. improving, low vs. middle, low vs. high, improving vs. middle, improving vs. high and middle vs. high, respectively

### Associations between ASBI performance during implementation/short-term sustainment phase (2014–2018) and ASBI performance during long-term sustainment phase (2019–2021)

Screening performance during 2014–2018 was significantly associated with screening rates 2019–2021. As shown in Table 2, screening rates during 2019–2021 for the low screening performance group continued to be significantly lower than the other two screening performance groups, while BI performance during 2014–2018 was not associated with screening rates 2019–2021. BI performance during 2014–2018 was significantly associated with BI rates 2019–2021, with all cross-BI group comparisons significant at *p* < 0.05 level, but screening performance during 2014–2018 was not significantly associated with BI rates 2019–2021.

**Table 2:** Screening and BI Rates during 2019–2021 by Screening and BI Performance Groups during 2014–2018

	Screening rates 2019–2021			BI rates 2019–2021		
	Mean	(95% CI)	*P* value	Mean	(95% CI)	*P* value
Screening performance 2014–2018			<.001			0.553
Low	69.27	(63.63, 74.91)		44.08	(34.01, 54.15)	
Middle	82.05	(78.03, 86.07)		45.29	(38.20, 52.37)	
High	85.83	(83.30, 88.35)		41.07	(36.65, 45.49)	
BI performance 2014–2018			0.858			<.001
Low	79.77	(76.12, 83.41)		22.13	(15.64, 28.61)	
Improving	78.80	(73.41, 84.19)		49.67	(40.18, 59.17)	
Middle	79.84	(75.68, 84.00)		37.16	(39.74, 44.58)	
High	77.78	(73.83, 81.74)		64.95	(58.04, 71.87)	

Separate multivariable mixed-effects models were fit for screening rate and BI rate during 2019–2021, each adjusted for time and the binary indicator for each patient panel characteristic at the year (panel size, proportions of Women, White, age ≥ 65 and Medicaid insured; neighborhood deprivation index and risk score among patient panel)

*BI* brief intervention, *CI* confidence intervals

Among the patient panel characteristics, having proportions of women above median was negatively, while having panel size above median was positively, associated with screening rates during long-term sustainment phase (77.09 [73.77, 80.41] vs. 81.01 [77.41, 84.61], *p* = 0.048 and 81.33 [77.86, 84.79] vs. 76.77 [73.54, 80.01], *p* = 0.025, respectively); panel size above median was also positively associated with BI rates during long-term sustainment phase (45.96 [40.30, 51.61] vs. 41.00 [35.64, 46.36], *p* = 0.058) (not shown).

### Associations between ASBI performance during implementation/short-term sustainment phase (2014–2018) and facility-level alcohol use outcomes during 2014–2021

We observed a decreasing trend in facility-level alcohol use outcomes during years 2014–2021. Among those screened, average rates of exceeding daily limits decreased from 8.3% in 2014 to 3.2% in 2021, and average rates of exceeding weekly limits decreased from 4.7% in 2014 to 3.5% in 2021, with average rates of exceeding both daily and weekly limits decreased from 1.8% in 2014 to 0.8% in 2021 (not shown). Facility-level average rates of having 5 + HED was 3.0% in 2014, and gradually decreased to 1.3% in 2021.

**Fig. 3:**
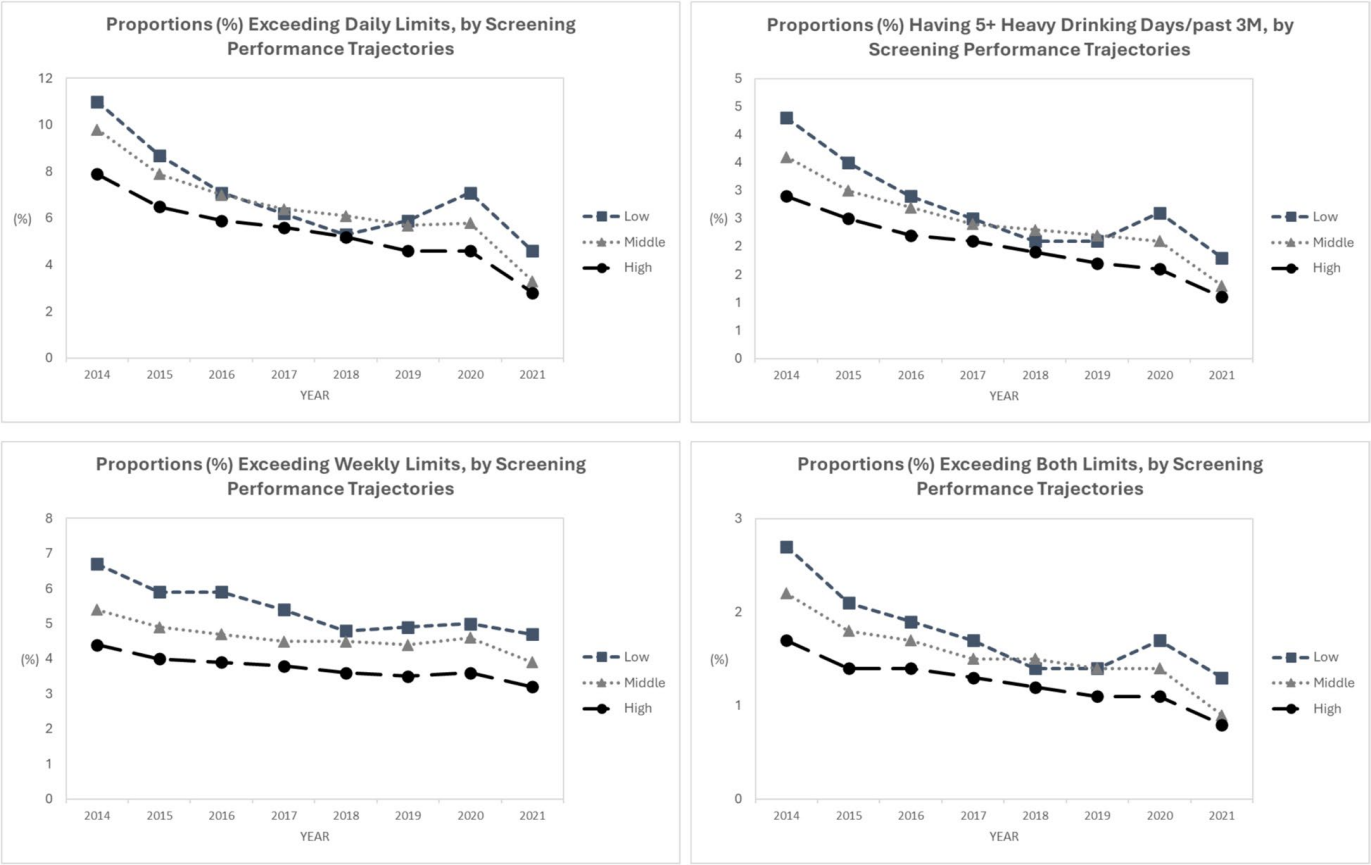
Alcohol Use Outcomes Over Time by Screening Performance Groups. Note. Separate multivariable mixed-effects models were fit for each of the alcohol use outcomes during 2014–2021, each included screening performance group 2014–2018 as the main independent variable, while adjusting for time (year) and the binary indicator for each patient panel characteristic at each year (panel size, proportions of Women, White, age ≥ 65 and Medicaid insured; neighborhood deprivation index and risk score among patient panel)

**Fig. 4:**
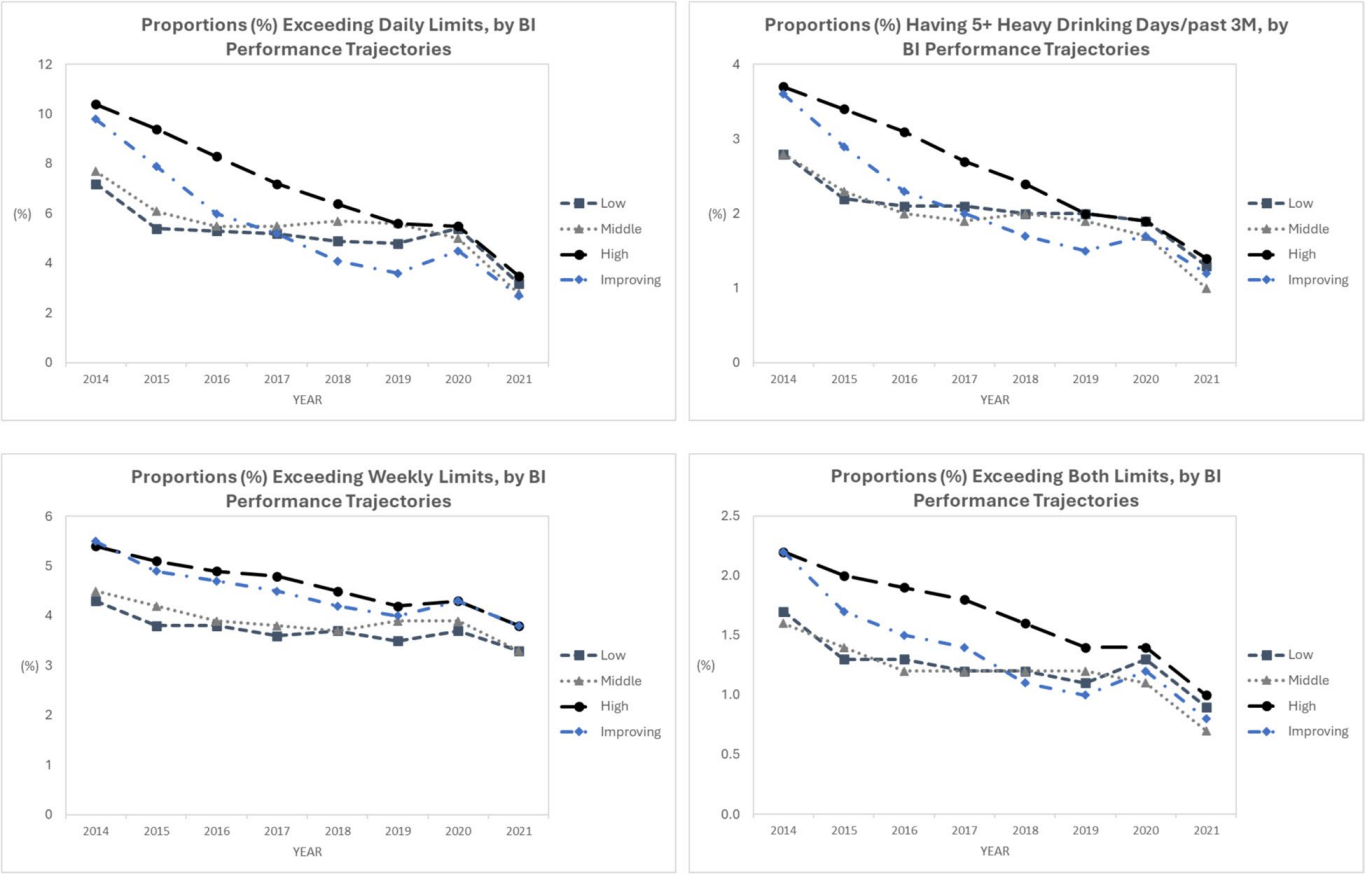
Alcohol Use Outcomes Over Time by BI Performance Groups. Note. Separate multivariable mixed-effects models were fit for each of the alcohol use outcomes during 2014–2021, each included BI performance group 2014–2018 as the main independent variable, while adjusting for time (year) and the binary indicator for each patient panel characteristic at each year (panel size, proportions of Women, White, age ≥ 65 and Medicaid insured; neighborhood deprivation index and risk score among patient panel). BI = brief intervention

On average, facilities in the high-screening-performance group 2014–2018 had the lowest adjusted prevalence rates for all four alcohol use outcomes 2014–2021. Differences across the three screening performance groups became smaller during 2017–2019 but then increased again in the 2020–2021 period, and there were significant between-group differences across years for proportions exceeding weekly limits (Fig. 3). While for all four alcohol use outcomes, p values were not statistically significant for either the main effect of BI performance group or the interaction with time, we observed a steeper decline in adjusted prevalence rates of reporting “exceeding daily drinking limits” and “having 5 + HEDs” over time among patients at facilities in the improving- and high-BI-performance groups (Fig. 4).

## Discussion

This study analyzed EHR data in a large, integrated healthcare system that implemented systematic ASBI in adult primary care in June 2013 and found variations in screening and BI performance trajectories during the implementation/short-term sustainment phase, defined as the first 5 years post implementation. In addition, we found that success in ASBI performance during the implementation/short-term sustainment phase was associated with long-term sustainability (6–8 years post-implementation). It suggests that poorer ASBI performance should be addressed early on rather than assuming that performance will improve over time.

Relatively greater variation was observed in BI performance than in screening across years. This is not a surprise given the overall success in screening performance in this healthcare system (post implementation, average screening rates increased and remained at above 91.0% before the COVID pandemic). For facilities in the middle and high screening performance groups, their average screening rates decreased somewhat but remained at or above 80% in years 2019–2021, during which the COVID-19 pandemic caused many disruptions of healthcare routines, resulting in a substantial reduction in alcohol screening and BI rates immediately after the onset of the pandemic. While 14% of the sites were in the “improving” BI performance group during the implementation/short-term sustainment phase, twice that many started out and remained low performing. Facilities in the improving and high BI performance groups continued to have higher BI rates during 2019–2021. Further research is needed to more closely examine what factors – system, organization, site and patient levels – are associated with the differences in ASBI performance as findings may have important implications for future SBIRT implementation and sustainment.

We found that the low BI performance group had panels of patients living in more deprived neighborhoods – a proxy indicator for lower socioeconomic status (SES) – as compared to the other three BI performance groups. A recent US study of a national sample of 44 primary care practices with co-located behavioral health services also found that higher practice-level social deprivation index, indicating more disadvantaged counties, was associated with lower odds of documentation of alcohol-related problems after adjusting for individual-level covariates [30]. Settings with lower SES patient populations may need to juggle more competing clinical priorities as patients present with more pressing needs and social risk factors. However, unhealthy alcohol use may have more profound and deleterious impacts on overall health in lower SES communities [31], and healthcare providers in those communities may need to be especially attuned to ASBI performance, and provide remedial training as needed. We did not find statistically significant associations between other patient panel characteristics and ASBI performance trajectories; however, non-White individuals have been found to have more consequences from similar or lower levels of alcohol consumption [32, 33]. The mechanisms of such findings remain to be fully identified and explained. Nonetheless, our findings suggest the need for systematic, public health approaches to provide support. Efforts may include tailored training and resources for care settings where populations might have greater unmet needs. Clinician BI training could also focus on incorporating information on alcohol consequences particularly salient to racially minoritized groups, such as disproportionate rates of and deleterious effects of chronic disease among some racially minoritized populations, and the association between chronic diseases and alcohol consumption.

The finding that larger panel size was related to better ASBI performance during long-term sustainment phase may seem counterintuitive, as one might expect lower performance with more patients per provider. However, larger panel size may be a proxy for facilities with more infrastructure and systemic resources, which have been found to be one of the most common facilitators of ASBI implementation and sustainment [34]. The finding that a higher proportion of women at sites was associated with lower screening performance during long-term sustainment phase is concerning and should be explored more thoroughly. Some previous research has found women more likely to report screening [35] but less likely to receive BIs [36–38]. This is of specific concern as there is a narrowing sex gap in alcohol use and related harms observed in the US [39–41]. It is important for systems to be aware of in order not to exacerbate disparities in addressing alcohol problems among women.

Findings from exploratory analyses suggest that early success in ASBI performance may also have a positive impact on population-level drinking outcomes longitudinally. This is encouraging and warrants future research. In view of the modest BI effects on alcohol use found in general medical settings [42] concerns have been raised that “[t]here is no longer support for the idea that brief intervention programmes alone can contribute meaningfully to the improvement of population health” [43]. Yet lack of systematic implementation in the U.S. outside of large integrated health systems limits evaluations of the long-term impact of ASBI on a population level. While not all findings were statistically significant, we observed a consistent pattern that over time, facilities in the middle and high screening performance groups had lower adjusted rates for all 4 alcohol use measures, and facilities in the improving and high BI performance groups had a steeper decline in adjusted rates of reporting “exceeding daily drinking limits” and “having 5 + HEDs” among their patient panels. Continued careful evaluations of the long-term impact of ASBI in real-world settings, including on alcohol-related health outcomes, and using patient-level data are needed.

### Limitations

This study has several limitations. Similar to other EHR-based studies, data on ASBI were limited to what was documented in the EHR. Alcohol use measures were based on self-report and subject to social desirability bias and possible underreporting. However, KPNC designed an alcohol screening workflow using evidence-based screening questions [44] and careful clinician training to minimize stigma and optimize patient comfort in disclosing alcohol use based on results of a trial [45]. As screening has become part of the regular clinical workflow and patients are re-screened, patients may have learned to under-report consumption in order to avoid repeated BIs. It is unknown what, if any, the effects of ASBI performance would have on patient reporting, and more research is warranted to examine the associations between different screening and BI performance trajectories and patient outcomes while properly accounting for this possible dynamic. Future research could also consider incorporating biomarkers, collateral reporting, or mixed methods to complement self-reported alcohol use data. KPNC has a well-established EHR and a diverse membership that reflects the U.S. population with access to care, allowing us to study a large population-based sample of patients and providers, yet it is not known how well the study’s findings may generalize to other healthcare systems and populations. Alcohol use outcomes were based on responses for patients who completed the screening. While the average screening rates have been high, the number of patients screened could vary between facilities and over time, which may affect how the results compared across different ASBI performance groups. Common to all observational data analyses, findings should not be interpreted as causal. Other programs (like patient education) during study period could have impacts on the observed trends in alcohol use.

### Study considerations and implications

KPNC is a well-resourced U.S. health system with a robust EHR, which has likely contributed to its successful ASBI implementation and sustainment. Other health systems and contexts may lack some of the factors which contributed to its success, but may have other facilitators factors which KPNC does not. Nevertheless, there are clearly many common and salient barriers and facilitators across these systems and findings from studies evaluating KPNC’s ASBI initiative in adult primary care, including those reported in the current study and previously published, add to the literature in understanding distinct trajectories of ASBI performance over time, the associated facilitators/barriers and patient panel characteristics, and potential population-level impact. Our findings that “early success in ASBI performance is associated with long-term sustainability and may also have a positive long-term impact on population-level drinking outcomes” could be applicable across many types of practices and systems. While the use of EHRs can facilitate alcohol screening, ASBI programs have long been conducted in the absence of EHRs, and are being implemented in lower-resource settings around the globe [46, 47]. Recommendations for ways to optimize implementation and increase access across variously resourced settings include clinical task-sharing, practice facilitation, thorough staff training, supporting implementation infrastructure, adopting collaborative care approaches in primary care, and using technology-enabled/virtual approaches.

## Conclusions

Early success in ASBI performance is associated with long-term sustainability and may be associated with long-term population-level drinking outcomes. Tracking clinic performance early and scaffolding implementation infrastructure through early and thorough clinician and staff training on the effectiveness and feasibility of ASBI, and provision of extra resources and assistance to underperforming clinics [23], especially those serving vulnerable patients, may be crucial for ASBI sustainability.

## Supplementary Information

Supplementary Material 1.

## Data Availability

The data that were used in the study were directly obtained from Kaiser’s EHR health system. Because of privacy issues, we are not allowed by the Kaiser Permanente Northern California Institutional Review Board to make these data publicly available. We are able to share metadata regarding the study upon request.

## Abbreviations

ASBI: Alcohol Screening and Brief Intervention
BI: Brief Intervention
HED: Heavy Episodic Drinking
HER: Electronic Health Record
KPNC: Kaiser Permanente Northern California
LCGA: Latent Class Growth Analysis
NDI: Neighborhood Deprivation Index
NIAAA: National Institute on Alcohol Abuse and Alcoholism
