# Supplementary material for: Trajectories of alcohol screening and brief intervention (ASBI) performance and their associations with long-term performance and alcohol use outcomes: an observational study in a large US integrated healthcare delivery system
Source: Implement Sci Commun. 2025 Dec 26;7:16. doi: 10.1186/s43058-025-00842-6 (PMC12849622; doi:10.1186/s43058-025-00842-6)
Supplement: Supplementary file 1 — Supplementary Material 1. [file 43058_2025_842_MOESM1_ESM.docx]

**Trajectories of alcohol screening and brief intervention (ASBI) performance and their associations with later performance and alcohol use outcomes**

**Supplementary Materials**

**Contents**

Supplementary Document. Protocol for the system-wide alcohol screening and brief intervention at Kaiser Permanente Northern California adult primary care………………………………….………………… 2-3

sTable 1. Measures of statistical fit across models, latent class growth analysis for screening performance during years 2014-2018….…...……………………………………………………………………….…… 4

sTable 2. Posterior probability of group membership by screening performance groups assigned to..…… 4

sTable 3. Measures of statistical fit across models, latent class growth analysis for BI performance during years 2014-2018…………………………………………………………………………………………… 4

sTable 4. Posterior probability of group membership by BI performance groups assigned to……….…… 4

**Supplementary Document. Protocol for the System-wide Alcohol Screening and Brief Intervention at Kaiser Permanente Northern California Adult Primary Care**

In June 2013, Kaiser Permanente Northern California (KPNC) implemented a program of systematic alcohol screening and brief intervention (ASBI) into its adult primary care workflow. Informed by the literature and by previous work of the research team,^1^ Alcohol as a Vital Sign (AVS) is a KPNC system-wide ASBI initiative in which medical assistants administer the screening, and physicians conduct the brief interventions and referrals to treatment, as needed.

**Screening.** In the AVS initiative, medical assistants conduct screening using National Institute on Alcohol Abuse and Alcoholism (NIAAA) evidence-based screening instruments^2^ embedded in the electronic health records (EHR) (using a modified version of the NIAAA screening question (“How many times in the past 3 months have you had 5 or more drinks in a day” [for men aged 18-65, and “4 or more drinks” for women and individuals aged 66 and older]), followed by two questions on typical drinking days per week and typical number of drinks per day). The medical assistants ask these questions as they collect vital sign information, and record patient answers in the EHR.

The EHR alerts medical assistants with a reminder to screen patients annually, except for those who had a prior positive alcohol screening, in which case the reminder is provided every six months until the patient has a negative screening.

**Brief Intervention (BI).** In addition to embedded screening instruments, the EHR provides clinical decision-support tools to aid BI delivery. Patient responses to the screening questions are displayed, and a “best practice alert” indicates to the physician if the patient is drinking over recommended limits. If the patient has screened positive, the EHR recommends delivering a brief intervention, provides further validated alcohol use disorder risk questions^3^ and suggests referral to the specialty alcohol treatment program for further assessment and treatment if problem acuity is high. Scripts for discussing unhealthy alcohol use and for facilitating referral to treatment are also provided in the EHR. Physicians are trained to deliver a BI (based on the NIAAA Clinicians Guide^2^ in which they state their concern and advise the patient to cut back to lower risk limits or abstain. Physicians are trained in Motivational Interviewing principles and to incorporate addressing salient presenting medical conditions (e.g., “drinking above these limits can worsen your hypertension…,” “cutting back from two drinks to one per night could help reduce your sleeping problems,” etc.), to ask patients how ready they were to make the recommended changes, and to assist in goal-setting to reduce or stop drinking if the patient was willing. A “train the trainers” model is used to disseminate the AVS protocols. These physicians at each facility, called “Alcohol Education Champions,” receive training adapted from the Alcohol Clinical Training (ACT) curriculum,^4^ and are responsible for training their colleagues and new physicians, and served as alcohol SBIRT experts for their facilities. Physicians receive a one hour in-person training from these Alcohol Education Champions, with supplemental training webinars and materials, and ongoing technical assistance and coaching.

**Referrals to Treatment.** Per protocol, if a patient screens positive, physicians offer a BI, and depending on level of consumption and problems acuity, make a referral to outpatient addiction medicine treatment for further assessment and treatment as needed.

**References**

1. Mertens JR, Chi FW, Weisner CM, et al. Physician versus non-physician delivery of alcohol screening, brief intervention and referral to treatment in adult primary care: The ADVISe cluster randomized controlled implementation trial. *Addict Sci Clin Pract* 2015;10(26):26. doi: 10.1186/s13722-015-0047-0 pmid: 26585638

2. National Institute on Alcohol Abuse and Alcoholism. Helping patients who drink too much: a clinician’s guide, 2005, updated 2007.

3. Vinson DC, Kruse RL, Seale JP. Simplifying alcohol assessment: two questions to identify alcohol use disorders. *Alcohol Clin Exp Res* 2007;31(8):1392-8. doi: 10.1111/j.1530-0277.2007.00440.x pmid: 17559544

4. Boston University School of Medicine/Boston Medical Center. Alcohol screening and brief intervention curriculum. Last updated July 12, 2007. https://www.bu.edu/act/mdalcoholtraining/index.html (accessed October 21, 2022).

sTable 1. Measures of statistical fit across models, latent class growth analysis for screening performance during years 2014-2018

|  | BIC | AIC | LL |
| --- | --- | --- | --- |
| 2-class | -826.41 | -808.34 | -798.34 |
| 3-class | -806.69 | -779.59 | -764.59 |
| 4-class | -820.72 | -784.59 | -764.59 |

Note: AIC=Akaike information criterion. BIC=Bayesian information criterion. LL=log likelihood.

sTable 2. Posterior probability of group membership by screening performance groups assigned to

|  | Low Screening Performance Group | Middle Screening Performance Group | High Screening Performance Group |
| --- | --- | --- | --- |
| Group 1 | **>.99** | <.01 | <.01 |
| Group 2 | <.01 | **.96** | .03 |
| Group 3 | <.01 | .04 | **.97** |

sTable 3. Measures of statistical fit across models, latent class growth analysis for BI performance during years 2014-2018

|  | BIC | AIC | LL |
| --- | --- | --- | --- |
| 2-class | -1155.12 | -1137.05 | -1127.05 |
| 3-class | -1138.37 | -1111.27 | -1096.27 |
| 4-class | -1141.35 | -1105.22 | -1085.22 |

Note: AIC=Akaike information criterion. BI=brief intervention. BIC=Bayesian information criterion. LL=log likelihood.

sTable 4. Posterior probability of group membership by BI performance groups assigned to

|  | Low BI  Performance Group | Improving BI Performance Group | Middle BI Performance Group | High BI  Performance Group |
| --- | --- | --- | --- | --- |
| Group 1 | **.96** | <.01 | .04 | <.01 |
| Group 2 | <.01 | **.97** | .05 | .06 |
| Group 3 | .04 | .02 | **.90** | .02 |
| Group 4 | <.01 | .01 | .01 | **.92** |

Note: BI=brief intervention.
